# Methylmercury Pause: Study Suggests Long Latency for Neurotoxicity

**Published:** 2008-06

**Authors:** Julia R. Barrett

Methylmercury (MeHg) easily crosses the blood–brain barrier and accumulates in the central nervous system, where it is demethylated to inorganic mercury. Chronic perinatal exposure to environmentally relevant levels of MeHg is associated with the occurrence later in childhood of neurobehavioral problems such as impaired attention and fine motor function. Animal studies confirm this association, but epidemiologic evidence is mixed despite extensive study. Moreover, MeHg toxicity and the period of time before effects appear are not completely understood, as few studies have been conducted beyond the first months or years of life in either animals or humans. Researchers now demonstrate in a mouse model that effects from early exposure to methylmercury can occur years after early-life mercury levels in the brain have declined **[*EHP* 116:746–751; Yoshida et al.]**.

In the current study, investigators used two strains of mice—the wild-type C57BL strain and the genetically manipulated metallothionein (MT)-null strain. The latter was used to examine potential genetic susceptibility to the toxic effects of MeHg exposure, as MT-null mice do not produce metallothionein-I and II proteins that can bind metals and protect against their toxic effects. Mice were exposed through diet to low levels of MeHg (5 μg/g diet) from the first day of pregnancy through the tenth day after birth. Offspring of the treated mice were weaned at 28 days. At 12 and 52 weeks (roughly comparable to young adulthood and middle age in humans), the offspring underwent behavioral tests of their locomotor activity and learning ability. All animals were weighed biweekly, and mercury concentrations in the brains, livers, and kidneys were measured for 10-day-old mice and for the group tested at 12 weeks.

In 10-day-old exposed mice, mercury concentrations in the brain were 0.5 μg Hg/g body weight or lower, with no significant differences observed between exposed wild-type mice and MT-null mice. At 13 weeks, concentrations of mercury in the brain of exposed groups were similar to those of the unexposed groups. Except for one activity measure in female MT-null mice, exposure to MeHg did not significantly affect behavioral test responses at 12 weeks. At 52 weeks, however, investigators observed significant effects in all behavioral test responses, with MT-null mice being slightly more affected. After 28 weeks, wild-type male and all MT-null mice exposed to MeHg weighed significantly less than control mice, which may signal an emerging toxic effect.

The authors demonstrate a long latency period after perinatal exposure to low levels of MeHg and show that this period may be influenced by genetic susceptibility, given the stronger effect of MeHg exposure in MT-null mice. The existence of a latency period suggests that a slow process, such as aging, plays a role in MeHg toxicity, although the actual damage occurs much earlier in life.

